# Smoking from a Younger Age Is the Dominant Factor in the Incidence of Chronic Obstructive Pulmonary Disease: Case-Control Study

**DOI:** 10.3390/ijerph18116047

**Published:** 2021-06-04

**Authors:** Winda Safitri, Santi Martini, Kurnia Dwi Artanti, Chung-Yi Li

**Affiliations:** 1 Faculty of Public Health, Universitas Airlangga, Surabaya 60236, East Java, Indonesia; winda.safitri@staf.unair.ac.id; 2 Department of Epidemiology, Faculty of Public Health, Universitas Airlangga, Surabaya 60115, East Java, Indonesia; kurnia-d-a@fkm.unair.ac.id; 3 Doctoral Program of Public Health, Faculty of Public Health, Universitas Airlangga, Surabaya 60115, East Java, Indonesia; 4 Department of Public Health, College of Medicine, National Cheng Kung University, Tainan 701, Taiwan; cyli99@mail.ncku.edu.tw; 5 Department of Public Health, College of Public Health, China Medical University, Taichung 404, Taiwan; 6 Department of Healthcare Administration, College of Medical and Health Science, Asia University, Taichung 413, Taiwan

**Keywords:** chronic obstructive pulmonary disease, tobacco use, initial smoking age, smoking habit, smoking duration

## Abstract

Background: Indonesia ranks 7th highest in the world for the number of deaths caused by tobacco use including those caused by Chronic Obstructive Pulmonary Disease (COPD). The purpose of this study was to determine the influence of initial smoking age and habit on the incidence of COPD. Methods: This research was a case-control study. The sampling in this research took a systematic random sampling method. The samples of this study were 56 respondents of a case group and 56 respondents of a control group. This study was conducted at Ngudi Waluyo Hospital, Wlingi, Blitar from October to November 2017. Results: The factors that influenced the incidence of COPD were being male (*p* = 0.00; OR = 6.333; 95%CI = 2.776–14.450), a smoker (*p* = 0.00050; OR = 5.1318; 95%CI = 1.9004–13.8958), initially smoking at <15 years old (*p* = 0.00; OR = 11,769; 95%CI = 4.086–33.903), initially getting into a smoking habit at the age of <15 years old (OR = 12; CI = 1346–106,950), initially getting into a smoking habit at the age of ≥15 years old (OR = 3647; CI = 1625–8183) and having smoked for ≥30 years (OR = 8857; CI = 3298–23,787). Conclusion: There are three factors of smoking behavior that influence COPD: smoking habit, initial smoking age and smoking duration. Of all factors, forming a smoking habit at the age of <15 years old has the biggest risk (OR = 12; CI = 1346–106,950).

## 1. Introduction

Chronic obstructive pulmonary disease (COPD) is a non-communicable disease with an incidence worldwide that increases from year to year [1]. COPD is a major source of morbidity, mortality and cost in the Western world [2]. Currently, the number of people with COPD globally reaches 384 million; it is estimated that this number will continue to increase, with COPD becoming the third-leading cause of death in the world by 2030 [3]. The burden of chronic respiratory diseases is generally increasing across the globe, and COPD is one of the main causes of mortality and morbidity [2].

The main risk factor for COPD is smoking [3]. Cigarette smoking has been shown to be the leading cause of COPD in the United States [4]. The pathogenesis of smoking-related COPD includes the protease, anti-protease and oxidant-antioxidant hypotheses and abnormal repair processes [5]. COPD caused by smoking is usually initiated by injury to the lungs. A smoking habit is the cause of eight out of ten cases of COPD [6]. More than 75% of COPD cases result from lung injury caused by a long period of smoking [7].

Other risk factors that can influence COPD are environmental exposure to biomass fuels and air pollution, and host factors such as age, sex or socioeconomic status [3,8]. Age is often listed as a risk factor for COPD as all vital organs lose their function with age, hence the decline in lung function, which occurs progressively after about 25 years of age. In the case of COPD, the age factor plays a role in increased cell aging, stem cell fatigue, increased oxidative stress, changes in the extracellular matrix and a reduction in endogenous anti-aging molecules and protective pathways such as autophagy [9]. In the past, most studies have shown that the prevalence and mortality of COPD are higher in men than women, but recent studies in developed countries have reported that the prevalence of COPD in men and women is almost the same. This may be due to changes in smoking behavior patterns [10].

COPD is the first cause of disability in the world [11]. Limited ability to perform daily activities occurs in three out of four cases of COPD [12]. The disease also limits a person’s ability to climb the stairs [13]. Early retirement occurs in 40% of patients with COPD [14]. In Europe, COPD accounts for 50% of total health funds each year and causes an annual productivity loss of €48.4 billion [15]. In the United States, it is estimated that COPD costs $50 billion each year, consisting of direct costs of as much as $30 billion and further indirect costs reaching $20 billion [16]. It is estimated that 12 million people suffer from COPD and that 120,000 die of the condition each year [17]. In Indonesia, COPD results in a total daily loss of productivity of 901,744 h and medical expenses of IDR 1,294,165,188,810; it is the sixth-leading cause of death nationally [18]. As such, it is necessary to make a preventive effort to reduce the prevalence of COPD. With that aim in mind, the purpose of this study is to determine the influence of initial smoking age and habit on the incidence of COPD.

Health is a human right mandated by the 1945 Constitution of the Republic of Indonesia; therefore, there is a need tobacco control for protection against the effects of cigarette smoke, especially for people who are not active smokers, in accordance with Health Law No. 36 of 2009 article 115 concerning the establishment of a No Smoking Zone (KTR) policy as part of government efforts to protect the public in the public environment. Based on 2013 health data, out of 38 districts/cities, only nine had implemented the KTR policy in East Java Province [19].

Blitar, a district in East Java, had still not implemented this regulation in 2018, despite one of the diseases caused by smoking, namely COPD, having a fairly high prevalence (3.7%) in the district compared to other diseases [19]. Based on a recapitulation of patient data from Ngudi Waluyo Wlingi Hospital, Blitar District, in 2016, the morbidity rate of COPD inpatients was ranked tenth with 226 patients, while for outpatient care, COPD was ranked thirteenth with 953 patients [18]. The main risk factor for COPD is exposure to cigarette smoke. Of the total population aged ≥10 years in Blitar District, 36.3% have a history of smoking habits, with 30% still actively smoking. In Blitar District, the average number of cigarettes smoked per person per day in 2013 was 9.7 cigarettes [19].

## 2. Material and Methods

### 2.1. Study Area

This study took place in Ngudi Waluyo Wlingi Hospital, Blitar District, Indonesia. Worldwide, Indonesia has the 7th highest number of deaths due to COPD, while Blitar district is one of the districts in East Java, Indonesia that does not have regulations regarding smoking-free areas. As a result, the prevalence of smoking-related diseases is high in Blitar District, one of which is COPD. The prevalence of COPD in Blitar District, East Java, Indonesia is 3.7% [19]. Ngudi Waluyo Wlingi Regional Hospital was designated as a Regional Public Service Agency (BLUD) Hospital in Blitar. It is a teaching hospital that has 16 accredited services and, as such, a suitable place for research. Based on a recapitulation of patient data from Ngudi Waluyo Wlingi Hospital, Blitar District, in 2016, the morbidity rate of inpatient COPD was ranked tenth with 226 patients, while for outpatient care, COPD was ranked thirteenth with 953 people [20].

### 2.2. Data Source

This research was an analytical observational study using a case-control design. This research was conducted in April–December 2017 at Ngudi Waluyo Hospital in Wlingi, Blitar, East Java, Indonesia. The case group was people who had been diagnosed as suffering from COPD, while the control group was people who had never been diagnosed as suffering from COPD.

### 2.3. Sample Selection

The sampling used in this study was systematic random sampling. The case population in this study was patients with COPD aged ≥30 years at Ngudi Waluyo Wlingi Regional Hospital, Blitar District, East Java. The diagnosis of COPD was determined based on the doctor’s diagnosis on the patient’s medical record. Meanwhile, the control population in this study were all patients aged ≥30 years who did not have a history of COPD based on medical records from Ngudi Waluyo Wlingi Regional Hospital, Blitar District, East Java. The sample size was calculated using Lemeshow’s comparison case, which generated 56 samples in each group (1:1). The samples were divided into case and control groups. The case group consisted of samples with COPD, while the control group consisted of samples without COPD. The cases and controls were selected randomly using a systematic random sampling technique. The control group was selected based on the current age of the respondent, namely, at least 30 years old. Systematic random sampling was carried out in both the case and control groups based on the serial numbers of patients seeking treatment at Ngudi Waluyo Hospital. This was based on the number of patients receiving treatment divided by the number of samples needed; the sample was taken based on the multiple of the results of the distribution in the sampling frame.

### 2.4. The Operational Definition of the Variable

The dependent variable studied was COPD, while the independent variables studied were divided into two groups: characteristics of respondents (age, sex, job and education) and smoking behavior (smoking habits, early smoking age and duration of smoking). Determination of COPD status was made in the case group using medical records and in the control group using a questionnaire. The age variable was determined based on the interview date and the respondent’s date of birth, and grouped into two categories: adult (≥30–<65 years old) and elderly (≥65 years old). The sex variable was divided into two categories: male and female. The education variable was divided into two categories: low education (from no school up to junior high school) and higher education (from senior high school to college).

The smoking habit variable was divided into three variables: non-smokers (never smoked), smokers (people who have smoked at least 100 cigarettes in their lifetime but in the environments where they live and work there are no smokers) and smokers who were exposed to secondhand smoke (smokers who are exposed to cigarette smoke itself and from other people). The variable of initial smoking age was divided into three groups: non-smokers, smokers who initially smoked at <15 years old and smokers who initially smoked at ≥15 years old. The variable of smoking duration was divided into three groups: non-smokers, smokers who smoked for <30 years and smokers who smoked for ≥30 years.

### 2.5. Instruments

Data collection was carried out using a questionnaire and secondary data, i.e., patient medical records. The questionnaire used in this study was a modification of that for basic health research and non-communicable disease research. The questionnaire was on participants’ smoking habits and statuses in relation to COPD. The questionnaire had been tested on 20 respondents to ensure its validity and reliability.

### 2.6. Statistical Analysis

The collected data were then analyzed descriptively and analytically with computer assistance. Data processing was carried out with bivariate analysis using the confidence of interval of 95% (α = 0.05).

### 2.7. Ethical Clearance

All participants were provided with written informed consent approved by the Ethics Commission of the Faculty of Public Health, Universitas Airlangga (certificate number: 536-KEPK).

## 3. Results

### 3.1. Characteristics of Respondents

Table 1 presents the distribution of respondent characteristics and their effects on the incidence of COPD, showing that of a total of 112 respondents, the majority of the COPD patients were male (75%), elderly (53.57%) and had a low educational background (67.86%). Significant results showed that males had a 6.33 times greater risk than females (*p* = 0.00; OR = 6.333; 95%CI = 2.776–14.450) and that the elderly group had an 11.769 times greater risk than respondents in the adult group (*p* = 0.00; OR = 11,769; 95%CI = 4.086–33.903).

### 3.2. Smoking Behavior

Table 2 presents the distribution of respondents’ smoking behavior and its effect on the incidence of COPD, showing that of a total of 112 respondents, 55 were smokers and 57 were non-smokers, with 29 of the latter exposed to secondhand smoke.

Significant results showed that smokers had a 5.1318 times greater risk of developing COPD compared with non-smokers (*p* = 0.00050; OR = 5.1318; 95%CI = 1.9004–13.8958). Yet, secondhand smokers had no influence on the incidence of COPD (*p* = 0.236; OR = 1.5278; 95%CI = 0.5028–4.6418). Significant results also showed that smokers who initially smoked at <15 years old had a 12 times greater risk of developing COPD compared with non-smokers (*p* = 0.026; OR = 12; 95%CI = 1.346–106.95), while smokers who initially smoked at ≥ 15 years old had a 3.647 times greater risk of developing COPD than non-smokers (*p* = 0.002; OR = 3.647; 95%CI = 3.298–23.787). A smoking period of fewer than 30 years did not influence the incidence of COPD (*p* = 0.881; OR = 1.091; 95%CI = 0.350–3.401), while a smoking period of more than 30 years was influential. Smokers who smoked for more than 30 years had an 8.857 times greater risk of developing COPD compared with non-smokers (*p* = 0.000; OR = 8.857; 95%CI = 3.298–23.787) (Table 2).

## 4. Discussion

### 4.1. Key Findings

The factors that influenced the incidence of COPD were being male (*p* = 0.00; OR = 6.333; 95%CI = 2.776–14.450), elderly (*p* = 0.00; OR = 11,769; 95%CI = 4.086–33.903), a smoker (*p* = 0.00050; OR = 5.1318; 95%CI = 1.9004–13.8958), developing an initial smoking habit at the age of <15 years old (*p* = 0.026; OR = 12; 95%CI = 1.346–106.95), developing an initial smoking habit at the age of ≥15 years old (OR = 3647; 95%CI = 1625–8183) and smoking for ≥30 years (OR = 8857; 95%CI = 3298–23,787).

### 4.2. Characteristics of Respondents

The current study showed that the OR value of the elderly group was higher than that of the adult group (*p* = 0.00; OR = 11,769; 95%CI = 4.086–33.903). This is in line with previous studies in Sousse, Tunisia in 2013 on elderly patients >70 years (*p* = 0.007; OR = 10,403; 95% CI = 2072–52,222) and in west and northern Sweden over 2008–2012 on elderly patients ≥60 years (OR = 8.40; 95%CI = 3.70–19.1), as well as based on the results of Indonesian health data from 2013 for elderly patients ≥60 years (OR = 4.5; 95%CI = 7.6–8.2) [21,22,23]. The three previous research studies have similar results, namely, that the elderly group has the highest risk of suffering from COPD versus other age groups.

The current study showed that the OR value of the male group was higher than for the female group (*p* = 0.013; OR = 3.273; 95%CI = 1.291–8.2999). This is in line with previous studies in Sousse, Tunisia in 2013 on a male group (*p* = 0.010; OR = 0.198 95%CI = 0.062–0.635) and in Anhui, China on a male group (OR = 2.01; 95%CI = 1.22–3.33), along with Indonesian health data from 2013 for a male group (OR = 1.3; 95%CI = 4–4.3) [21,22,24,25]. The four previous research studies have similar results, namely, that males have a higher risk of suffering from COPD than females. The differences in the ORs in each study, both for age and sex, could be caused by several factors, such as differences in the methods used and numbers of samples taken, and bias resulting from other influential risk factors.

### 4.3. Smoking Behavior

#### 4.3.1. Smoking Habit

The current study shows that the OR values for smokers are higher than those for other groups (*p* = 0.00050; OR = 5.1318; 95%CI = 1.9004–13.8958). This is in line with a previous study in 2019 in the Abeshge District, Southern Ethiopia on a smokers group (OR = 4.19; 95%CI = 2.59–6.78) [8]. Two previous studies in Sousse, Tunisia and Anhui, China, which described the smoking group in more detail, showed that current smokers have a higher risk than former smokers [21,25]. When compared with three previous studies, the OR value of a smoking habit in this study was higher than in those before. This may be because the methods used in this study were different and the number of participants was smaller. However, there was a similarity in that the smoker group had a greater risk of suffering from COPD than the non-smoker group. Smoking cigarettes over a long period disrupts ciliary movement, inhibits alveolar macrophage function and ultimately, leads to hypertrophy and hyperplasia of the mucus excreting gland [26]. Cigarette smoke contains many species of reactive oxygen (free radicals), which deplete the antioxidant mechanism, resulting in tissue damage and potentially leading to COPD [27].

#### 4.3.2. Initial Smoking Age

Developing a smoking habit at the age of <15 years old presents the biggest risk, more so than any other (OR = 12; 95%CI = 1346–106,950). There is a significantly higher risk of COPD when starting smoking at <15 years of age (3.647–12%) than ≥15 years old as smoking in childhood and adolescence can slow the growth and development of the lungs, thereby increasing the risk of incidence of COPD during adulthood. The lungs still grow and develop until the age of 20 years; therefore, smoking prior to the age of 20 years causes one’s lungs to develop sub-optimally both in terms of ability and function. These kinds of lungs fail to work properly, produce breath that tends to be short and will cause problems when used to exercise or perform physical activity. Although the health of people who quit smoking will increase dramatically compared with their health when they smoked, some cases of lung damage at an early age due to cigarettes are irreversible [28]. Therefore, it is necessary to implement policies regarding age restrictions on the purchase of cigarettes, especially for mobile cigarette sellers, as well as fostering cross-sector cooperation between health, education and other government sectors to spread the message about the dangers of smoking from an early age.

#### 4.3.3. Smoking Duration

Smokers who smoked for more than 30 years had an 8.857 times greater risk of developing COPD compared with non-smokers (*p* = 0.000; OR = 8.857; 95%CI = 3.298–23.787). Yet, the results for smoking <30 years were not in line with other studies, which showed that smoking has an effect, i.e., that someone who has had a smoking habit for >20 years is three to four times more likely to develop COPD compared to someone whose has had a smoking habit for ≤20 years [29]. The result of this study may have been influenced by exposure to cigarette smoke from other people, as of the 55 respondents who smoked, 26 were also exposed to cigarette smoke from other people, which could have disturbed the results of the study. Nonetheless, the results for smoking ≥30 years are in line with other literature stating that the longer the smoking habit, the greater the risk of developing COPD [30].

## 5. Conclusions

A starting age for smoking of less than 15 years old has the highest risk (OR = 12; 95%CI = 1346–106,950) for COPD. The other factors that influenced the incidence of COPD were being male (*p* = 0.00; OR = 6.333; 95%CI = 2.776–14.450), elderly (*p* = 0.00; OR = 11,769; 95%CI = 4.086–33.903) and with a smoking duration ≥ 30 years (OR = 8857; 95%CI = 3298–23,787).

## Figures and Tables

**Table 1:** Characteristics of Respondents.

Variables	Case		Control		*p*	OR	95% CI
	n	%	n	%			
**Sex**							
Male	42	75.00	18	32.14	0.00	6.333	2.776–14.450
Female	14	25.00	38	67.86			
**Age**							
Elderly (≥65 years old)	30	53.57	5	8.93	0.00	11.769	4.086–33.903
Adult (≥30–<65 years old)	26	46.43	51	91.07			
**Education**							
Low Education	38	67.86	36	64.29	0.69	1.173	0.536–2.567
High Education	18	32.14	20	35.7			
Total	56	100.00	56	100.00			

**Table 2:** Smoking Behavior of Respondents.

Variables	Case		Control		*p*	OR	95% CI
	n	%	n	%			
**Smoking Habit**							
Non-Smokers	8	14.28	20	35.72			
Secondhand Smokers	11	19.64	18	32.14	0.236	1.5278	0.5028–4.6418
Smokers	37	66.07	18	32.14	0.00050	5.1318	1.9004–13.8958
**Initial Smoking Age**							
Non-Smokers	19	33.93	38	67.86			2.599–13.620
Smokers who initially smoked at <15 years old	6	10.7	1	1.79	0.026	12	1.346–106.95
Smokers who initially smoked at ≥15 years old	31	55.4	17	30.35	0.002	3.647	1.625–8.183
**Smoking Duration**							
Non-Smokers	19	33.93	38	67.86			
Smokers who smoked for <30 years	6	10.71	11	19.64	0.881	1.091	0.350–3.401
Smokers who smoked for ≥30 years	31	55.36	7	12.5	0.000	8.857	3.298–23.787
Total	56	100.00	56	100.00			

## Data Availability

The data presented in this study are available on request from the corresponding author. The data are not publicly available due to some of the data taken contains the respondent’s personal information which cannot be disseminated publicly.

## Abbreviations

The following abbreviations are used in this manuscript:

COPD	Chronic Obstructive Pulmonary Disease
USD	United States Dollar
BLUD	Regional Public Service Agency (in English)
OR	Odds Ratio
CI	Confidence Interval
